# Tracing conformational changes in proteins

**DOI:** 10.1186/1472-6807-10-S1-S1

**Published:** 2010-05-17

**Authors:** Nurit Haspel, Mark Moll, Matthew L Baker, Wah Chiu, Lydia E Kavraki

**Affiliations:** 1 Department of Computer Science, Rice University, Houston, TX 77005, USA; 2 National Center for Macromolecular Imaging, Verna and Marrs McLean Department of Biochemistry and Molecular Biology, Baylor College of Medicine, Houston, TX 77030, USA; 3 Graduate Program in Structural and Computational Biology and Molecular Biophysics, Baylor College of Medicine, Houston, TX 77030, USA; 4 Department of Bioengineering, Rice University, Houston, TX 77005, USA; 5 Currently with the Department of Computer Science, the University of Massachusetts Boston, Boston MA 02125, USA

## Abstract

**Background:**

Many proteins undergo extensive conformational changes as part of their functionality. Tracing these changes is important for understanding the way these proteins function. Traditional biophysics-based conformational search methods require a large number of calculations and are hard to apply to large-scale conformational motions.

**Results:**

In this work we investigate the application of a robotics-inspired method, using backbone and limited side chain representation and a coarse grained energy function to trace large-scale conformational motions. We tested the algorithm on four well known medium to large proteins and we show that even with relatively little information we are able to trace low-energy conformational pathways efficiently. The conformational pathways produced by our methods can be further filtered and refined to produce more useful information on the way proteins function under physiological conditions.

**Conclusions:**

The proposed method effectively captures large-scale conformational changes and produces pathways that are consistent with experimental data and other computational studies. The method represents an important first step towards a larger scale modeling of more complex biological systems.

## Background

**Figure 1:**
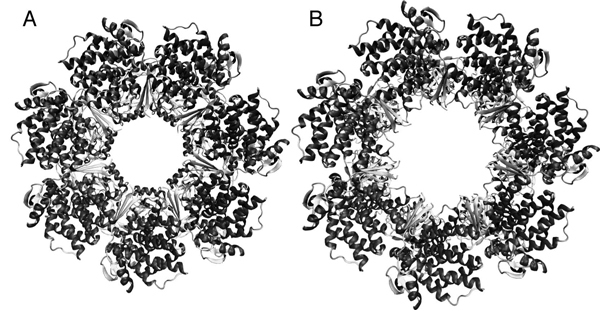
**GroEL** (a) The GroEL complex (PDB structure 1ss8). (b) The GroEL-GroES-ADP7 complex (PDB structure 1sx4).

Proteins are flexible molecules that undergo conformational changes as part of their interactions with other proteins or drug molecules [1]. Changes in torsional angles may induce localized changes or large scale domain motions. Figure 1 shows an illustration of the closed structure of the GroEL 7-membered single ring complex taken from PDB code 1SS8 (Figure 1(a)) and the opened structure (GroEL-GroES-ADP7) taken from PDB code 1SX4 (Figure 1(b)). GroEL transitions between the closed and open conformations as part of its chaperone activity, but the structural details of the transition process are not fully understood. Tracing these changes is crucial for understanding the way these proteins perform their function. Existing physics-based computational methods that trace and simulate conformational changes in proteins include Molecular Dynamics (MD) [2], Monte Carlo (MC) [3] and their variants. These methods require large amounts of computational resources and are therefore hard to apply to conformational motions that take place over time scales larger than several hundreds of nanoseconds. In the past two decades several efficient conformational search algorithms have been developed. Some use a coarse representation of the protein molecule [4-6] and employ various efficient search methods such as Normal Mode Analysis (NMA) [7,8] elastic network modeling [9-14], or morphing [15,16]. In recent years sampling based motion planning methods have been successfully applied towards an efficient exploration of protein conformational space. Motion planning is an area in robotics concerned with finding a pathway for robot-like objects in constrained environments [17-19]. When applied to biological problems, the protein is represented as an articulated body with the degrees of freedom in all or selected torsional angles. The physical constraints are implicitly encoded in a penalty function which approximates the potential energy of the molecule. The conformational space of the protein is explored so that high energy regions are avoided and feasible conformational pathways are obtained more efficiently than with traditional simulation methods. Among the many applications of motion planning to biology are the characterization of near-native protein conformational ensembles [20], the study of conformational flexibility in proteins [21,22], protein folding and binding simulation [23-25], modeling protein loops [21,26], simulation of RNA folding kinetics [27] and recently the elucidation of conformational pathways in proteins, subject to pre-specified constraints [28]. The search methods described above strike a balance between accuracy and efficiency. Many of those methods are successful in sampling the conformational landscape of proteins but are often biased by the protein native conformation and some of them require additional, problem specific information. Additionally, when atomic details are skipped the conformational search process is greatly accelerated but fine details are missed.

In this work we present a prototype of a novel, efficient motion-planning based methodology to perform conformational search on proteins requiring only backbone and limited side-chain information. The molecule is mapped into a reduced representation using a small number of parameters that represent its degrees of freedom. This allows for larger motions to be explored efficiently. We aim to make the conformational search as general as possible so it can be applied with as little system specific knowledge as possible. We use a coarse-grained physics based energy function which captures low energy conformations in a realistic but efficient way [29]. We identify the flexible parts of the proteins and manipulate them to simulate the conformational changes, treating the rest of the protein as rigid. In this way we reduce the dimensionality of the search space while still capturing the essential conformational flexibility of the protein. We tested our methodology on four proteins ranging in size from 101 to 525 residues that are known to undergo extensive conformational changes. The results show that we are able to efficiently produce low energy pathways for each one of them. The method can serve as a filtering tool which can provide biologists with useful hypotheses about the way proteins transition from one conformational state to another, and help to gain more insight about protein function.

### Problem statement

Given two conformational states of a molecule, denoted by start and goal, our objective is to find conformational pathways connecting the start and goal conformations. A pathway is a sequence of affine transformations that, when applied successively to the degrees of freedom of the start conformation, the start conformation will be brought to within a tolerance range of the goal conformation under a defined distance metric. Furthermore, the energy of each intermediate conformation along the pathway must be lower than a given threshold as measured by a potential function that approximates the protein energy. The degrees of freedom of the structures lie in the flexible parts connecting rigid structural elements. Several assumptions are made in this paper. We assume that secondary structure elements do not change significantly during domain motion and that the flexible parts are the loops connecting secondary structure elements. While this assumption is true in many cases, there are cases where secondary structure elements melt or change. In these cases, it is possible to incorporate a more detailed modeling of the flexible parts into the general framework of the algorithm without limiting the proposed procedure. It should be emphasized that the algorithm does not always produce the same conformational pathway, but rather a possible pathway. This is due to randomness in the search algorithm (see Methods section below). By repeating the procedure a large number of times we produce a set of feasible pathways, thus limiting the huge search space to a manageable number of possibilities. These pathways can later be clustered, refined and filtered using information about the tested systems. The size of the clusters can give us information about the likelihood of given conformations along the pathway.

## Results and discussion

Below we first describe in depth the conformational search method including the data structures, distance metric, energy function and search algorithm used to perform the conformational search and produce low-energy pathways. After the description of the method, we will present simulation results for four well-studied proteins.

### Data representation

We use a coarse grained representation used successfully by our research group in the past [29]. The proteins are stripped of their side-chain and hydrogen atoms and represented at the backbone and C_*β*_ level (Glycine is represented by its backbone only). The amino acids are grouped into secondary structure elements. The secondary structures can be assigned by the PDB header or using a secondary structure assignment algorithm such as DSSP [30]. Loop residues are assigned to the nearest secondary structure element. To save computational time, it is possible to cluster several secondary structure elements into one rigid element if their positions are known not to change with respect to one another during the conformational transition. Alternatively, to gain accuracy, in highly movable regions of the protein such as flexible loops or if some secondary structure elements are known to break or change, their structural representation can be refined and broken down to smaller sub-structures. This refinement is not considered in the context of this paper but it can be applied without loss of generality. The high-level data structure that represents a conformation is a graph *G* = (*V*, *E*) such that each secondary structure element is a node *v* ∈ *V* in the graph. Two secondary structure elements v_1_ and v_2_ are connected by an edge *e* ∈ *E* if there is at least one pair of adjacent amino acids* r_1_*,*r_2_*, such that *r_1_* ∈ *v_1_* and *r_2_* ∈ *v_2_*. The backbone angles in *r_1_*, *r_2_*, and a small number of sequentially adjacent residues form the degrees of freedom of the protein. In other words, the protein motions consist of bond rotations in these residues while the remaining angles stay fixed.

Based on the graph we construct a spanning tree* T* = (*V*, *X*) where *X* is a subset of *E* using a greedy approach. The root of the tree is specified as the structure that is expected to move the least during the search as determined by aligning the start and goal structures and measuring the least RMSD between corresponding secondary structure elements. Each one of the root's neighbors forms a child node in the tree, and at each stage the selected node and its adjacent edges are removed from the graph. The process repeats iteratively until all the secondary structure elements are represented in the tree. In some cases we may know that the poses of certain secondary structure elements is likely to stay fixed. This allows us to speed up the search for a feasible pathway by restricting motions to the remaining secondary structure elements. Let* K* ⊆ *V* be the set of secondary structures that is free to move. This set is used below in the definition of a distance metric for our representation.

### Handling symmetric complexes

Many biomolecules self-assemble into symmetric complexes. The GroEL chaperonin, described in more detail in the results section, is an example of such a symmetric complex. Each of its two rings contains 7 monomeric subunits. We imposed exact symmetry by using one monomer and applying the appropriate symmetry transformation. The symmetry is exploited in distance and energy calculations to improve computational efficiency of the conformational search. For distance calculations we can limit ourselves to distances between conformations of one monomer rather than the entire complex. Energy is computed similarly, except that care should be taken of the interaction energy between adjacent monomers.

### Distance between structures

Motion planning methods need a distance measure to estimate the progress of the search. In our representation of protein structures, there is not necessarily one-to-one mapping between atoms or residues in different conformations. Even if there was one, defining a distance measure over residue positions would be needlessly computationally expensive, since our algorithm will manipulate proteins at the secondary structure level. Below we will describe a distance measure defined in terms of the relative positions between secondary structure elements.

Given a conformation* C*, we first define a score for each secondary structure element *i* in* C*:

 (1)

The summation is over the set *K* of 'mobile' secondary structures in *C* excluding *i*,* α_ij_* is the angle and *d_ij_* is the distance between secondary structure element *i* and secondary structure element *j* in *C*,  is the angle and  is the distance between the corresponding secondary structure elements in the goal structure, and *w_i_* and   are weight factors proportional to the size of secondary structure element i, such that the angle and distance components will be brought to the same order of magnitude. In the current implementation we use the values of 1 for *w_i_* and 5 for , which seem to give the best results. An angle between two secondary structure elements is defined as the angle between the two vectors representing them. A vector representing a helix is the least-square straight line that passes through the helix atoms, and a vector representing a sheet is the normal to the plane best representing the sheet. The distance between two secondary structure elements is defined as the distance between their centers of masses. We then compute for a conformation *C* a feature vector:

 (2)

where the components of the vector are the scores of the* K* secondary structure elements of the conformation.

If the molecule is a complex, the score also measures the distances and angles between secondary structure elements from adjacent units, so that equation (1) above also contains terms from secondary structures on different symmetric units. To save computational time and due to the fact that distant monomeric subunits do not interact in a complex, we only included interactions between secondary structures taken from adjacent monomers.

The distance between two conformations,* C*_1_ and* C*_2_ is defined as the Euclidean distance between their feature vectors, i.e., . By definition, when *C*_2_ is the goal structure, the* score* of *C*_1_ is the magnitude of its vector representation. Therefore, the lower the score for a given conformation, the more similar it is to the goal structure. The feature vector is used as a projection of the conformation to a lower dimensional subspace that is used to measure coverage of the search space by the search method described below. It should be noted that other distance measures exist for our representation of protein structures [31], but after extensive experimentation the measure described above produced good results.

### Energy function

In order to approximate the potential energy of the produced conformations we suggest a simplified energy function which includes the following components:

*E*_total_ = *E*_soft-vdW_ + *E*_HB_ + *E*_burial_ + *E*_water_ + *E*_bond_ + *E*_angle_    (3)

The first four terms in this coarse-grained energy function are a part of an energy function successfully used in our group in the past [29]. The compaction term mentioned in [29], which biases the energy towards folded, compact structures, was removed from our implementation since we are not simulating protein folding. The bond and angle terms are taken from the AMBER ff03 force field [32]. If the structural manipulation causes the energy to be at least 100 kcal/mol higher than the energy of the starting structure 20 minimization steps are performed over the bond, angle and van der Waals energy terms of the manipulated secondary structure elements using a steepest descent scheme [33].

### Search methodology

The search is performed using a sampling-based motion planning algorithm. Motion planning algorithms have been applied extensively in the past to solve biological problems due to the analogy between protein chains and robotic articulated mechanisms [23-25]. The search methodology applied in this paper is based on the Path-Directed Subdivision Tree (PDST) planner [34-36]. We chose this algorithm because of its good performance with articulated systems with complex dynamics moving in physically constrained environments. We adapted the algorithm to model protein motions. In our adaptation, the planner iteratively constructs a tree of conformational pathways as the search progresses. The input to the algorithm consists of the start and end conformations of a molecule, represented as sets of articulated secondary structures as discussed in Data Representation above. The root of the search tree is a "pathway" of length 0 consisting only of the starting structure. At every iteration a previously generated pathway is selected for propagation using a deterministic scoring scheme described below. From a random conformation along that pathway, a new pathway is propagated by applying a small random rotation to the φ or ψ backbone dihedral angle of a residue that resides on a loop connecting two randomly chosen secondary structure elements. A molecular motion is sampled by applying the rotation until a high energy conformation is reached. The coarse grained energy function described above is used to determine when a high energy conformation is encountered. A high energy conformation is defined as being more than 50 kcal/mol above the starting energy. It should be noted that our search aims to cover the conformational space and simulate a pathway from the start towards the goal conformation. It is not a minimization scheme and therefore is not aimed towards the minimum energy conformation. This makes it suitable for cases where the goal conformation has a higher potential energy than the start conformation. The algorithm maintains a subdivision of the low-dimensional projection of the conformational space (described in Distance Between Structures above) into cells, such that no sample spans more than one cell in the subdivision. The goal of the subdivision is to guarantee coverage of the search space [35]. After a sample is selected for propagation, the cell containing that sample is subdivided into two cells. The algorithm keeps track of how many samples are contained in each cell to estimate how dense the sampling is in different areas of the space. It maintains a scoring scheme that gives selection preference to samples residing in large, empty cells, thus pushing the exploration towards unvisited areas in the conformational space. Probabilistic completeness is obtained via a scoring scheme that favors the selection of samples contained in larger cells and leads to unexplored areas of the search space. The sample scores are updated in a way that guarantees that every sample in the tree will eventually be selected for propagation and avoids over-sampling of parts of the space. A previous study in path-directed motion planning algorithms [37] showed that employing a biasing scheme in a small percentage of the iterations greatly improves the performance of the planner. Motivated by these results [37], we employed biasing at 10% of the iterations. During these iterations the scoring scheme described above is ignored and a sample is chosen out of a pool of conformations closest to the goal conformation, which gives the planner a better chance to successfully terminate the search. We found that the biasing improves the performance of the algorithm. Our top-level algorithm runs PDST iteratively. Each iteration runs until a generated conformation is closer to the goal conformation than a pre-specified intermediate distance threshold, where the distance threshold is determined by the distance measure described above. We found that a threshold of 0.8-0.9 of the distance between the start and goal conformations is usually sufficient to achieve good results. The iterative runs of the PDST planner help reduce memory use and improve performance, as also shown in [38]. To produce the results shown in this paper, three PDST cycles, each of 20,000 iterations, were allowed per run of the algorithm for each example.

### Simulation results and validation

We ran the PDST-based search algorithm described above on several test cases: Adenylate Kinase (AdK), Ribose binding protein (RBP), the 2 ring GroEL complex and Cyanovirin-N (CVN). These proteins have been chosen for the following reasons: all undergo extensive conformational transitions, they are well studied and have an abundance of data for testing and comparison.

For comparison purposes, we produced conformational pathways using a random walk using a Monte Carlo like algorithm [3]. In order to make the two methods as comparable as possible, we used the same representation, similarity score and potential function described in our algorithm. The random walk algorithm differs from the common use of Monte Carlo in protein conformational search. Rather than optimizing the energy, it optimizes the similarity score (see Distance between Structures subsection under Methods for definition) in order to simulate a conformational pathway from the start to the goal conformation. The energy, while not optimized, is used to filter out non-feasible conformations. The random walk implementation uses the Metropolis criterion for the selection of steps. At each iteration a random conformational pathway is generated from the current conformation by applying a small random transformation to either the φ or ψ dihedral angle of one of the degrees of freedom connecting secondary structure elements, in a similar way to the one used to generate new conformations described in the Search Methodology subsection above. If a step brings the similarity score of the generated conformation closer to the goal it will be accepted. Otherwise it is accepted with a probability proportional to *e*^Δ^*^S^* where Δ*S* is the difference in the similarity score of the current step and the previous step. In practice, this criterion accepts all "good" steps while allowing a very small fraction of "bad" steps.

In order to compare the performance of the two methods by an objective standard, each algorithm was run a 100 times per example and the least RMSD (lRMSD) of the closest conformation to the goal at that given time step was measured. lRMSD is the root mean square deviation between two conformations after alignment. In our implementation, only C_α_ atoms were considered for the lRMSD measurement. lRMSD was measured after 1 hour, 2 hours, and at the end of the run. All runs were allowed to continue for a maximum of 8 hours or until a generated conformation is closer to the goal structure than a specified threshold, varying according to the tested protein. All experiments were run on the Rice Cray XD1 Cluster, where each node runs at 2.2 Ghz and has 8 GB RAM. Table 1 summarizes the lRMSD statistics over 80 of the 100 test runs for each algorithm and protein test case, with the top and bottom 10% outliers excluded from the calculation.

**Table 1:** Performance statistics for the AdK, RBP, CVN and GroEL complex examples

	**AdK**	**AdK RW^†^**	**RBP**	**RBP RW^†^**	**CVN**	**CVN RW^†^**	**GroEL**	**GroEL RW^†^**
				
Initial lRMSD (Å)	6.95	6.95	4.06	4.06	16.01	16.01	14.64	14.64
#Residues	214	214	271	271	101	101	525^‡^	525^‡^
lRMSD after 1 hour (Å)	2.69±0.21	3.81±0.49	1.48±0.25	2.35±0.52	4.52±0.73	5.28±1.59	5.67±0.67	8.21±1.93
lRMSD after 2 hours (Å)	2.55±0.2	3.68±0.46	1.34±0.21	2.23±0.45	3.842±0.79	5.09±1.52	5.04±0.42	7.66±2.04
Final lRMSD (Å)	2.53±0.2	3.65±0.47	1.26±0.15	2.22±0.49	3.18±0.34	4.88±1.44	4.67±0.36	6.11±1.9

The average ± (standard deviation) lRMSD data were taken over 80 runs where the top and bottom 10% outliers were removed from the original set of 100 runs.

^†^ Random walk. See Results section for a discussion.

^‡^ This is the number of residues per monomer. As explained in the text, the symmetry is exploited to model the entire 7-ring complex, which has 7 x 525 = 3675 residues.

#### Adenylate kinase (AdK)

**Figure 2:**
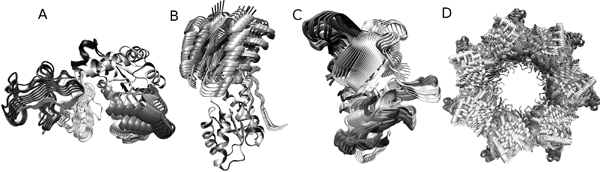
**Conformational pathways** Illustration of the results for AdK (a), RBP (b), CVN (c) and GroEL (d): The conformational pathways are obtained after side chain completion and basic energy minimization. The conformation colors are interpolated on the white (start) to black (goal) scale.

AdK is a monomeric phosphotransferase enzyme that catalyzes reversible transfer of a phosphoryl group from ATP to AMP. The structure of AdK, which contains 214 amino acids, is composed of the three main domains, the CORE (residues 1–29, 68–117, and 161–214), the ATP binding domain called the LID (residues 118–167), and the NMP binding domain (residues 30–67). AdK assumes an "open" conformation in the unligated structure and a "closed" conformation. The lRMSD between the two structures is 6.95Å. Supposedly, during the transition from the "open" to "closed" form, the largest conformational change occurs in the LID and NMP domain with the CORE domain being relatively rigid. Our model contains 10 rigid elements where most of the CORE domain was modeled as one large segment and was considered fixed, since it does not undergo a large-scale motion. The distance measure threshold for successful termination of the algorithm was a conformation with a distance of 0.16 from the goal conformation when the distances are normalized on a scale of 0–1 (the start conformation has distance 1 to the goal conformation). The threshold was chosen as a compromise between low RMSD and a reasonable runtime and memory consumption. Figure 2(a) shows an example of a pathway from the start to the end conformation. The C_α_ RMSD from the goal structure is 2.07Å. As seen in table 1 the resulting average lRMSD was 2.53Å. Random walk performed significantly worse compared to our planner with an average lRMSD of 3.65Å. The average running time was 3 hours, 58 minutes.

#### Ribose binding protein (RBP)

RBP is a sugar-binding bacterial periplasmic protein whose function is associated with large conformational changes upon binding to ribose. It is a 271 residue protein made of two domains, the first containing residues 1–99 and 238–260 and the second containing residues 104–233. The domains are linked by a three stranded hinge spanning residues 100–103, 234–237, and 261–271. The lRMSD between the two conformations is 4.06Å. We modeled the closed state to open state motion using PDB codes 2DRI and 1URP for the closed and open states, respectively. Our model contains 3 rigid elements where most of the N- and C-terminal domains were modeled as rigid segments and the hinge was modeled as a separate domain. The distance measure threshold for successful termination of the algorithm was a normalized distance of 0.08 from the goal conformation. As seen in table 1, the resulting average RMSD was approximately 1.38 Å. Random walk performed poorly comparing to our planner and the average RMSD in the end of the run was 2.59Å. In this example, as well as the AdK example above, the vast majority of the progress was achieved during the first 60 minutes of the run. Figure 2(b) shows an example of a pathway from the start to the end conformation. In this example, the C_α_ RMSD from the goal structure is 0.76Å. The average run time for our method was approximately 1 hour and 40 minutes.

#### Cyanovirin-N (CVN)

CVN is an anti-viral fusion inhibitor protein that binds to viral sugars, and is trialed for preventing sexual transmission of HIV. It comprises two repeat domains of 30% sequence identity. The domain swapped dimer has higher anti-viral affinity than the monomer [39], and it was shown that the two forms can exist in solution, with a high energy transition barrier between them. In addition, it has been reported that certain mutations can affect the energy barrier and stabilize alternative conformations [40]. We simulated the unpacking of the repeat domains of a single chain from the intertwined monomeric conformation to an extended domain-swapped conformation. The swapped conformations deviate by approximately 16Å. CVN contains 101 amino acids and our model contains 6 rigid elements. The flexible rotation axis resides mainly between residues 48-55. The distance measure threshold for successful termination of the algorithm was a normalized distance of 0.13 from the goal conformation. Figure 2(c) shows an example of a pathway from the start to the end conformation. The C_α_ RMSD from the goal structure is 2.06Å. As seen in Table 1, our algorithm significantly outperformed random walk with an average lRMSD of about 3Å comparing to nearly 5Å for random walk. Many of our runs got as low as less than 2Å from the final conformation. The average run time was approximately 2.5 hours.

#### GroEL complex

The GroEL protein belongs to the chaperonin family and is found in a large number of bacteria [41]. It is required for the correct folding of many proteins. GroEL requires the lid-like cochaperonin protein complex GroES. Binding of substrate protein, in addition to binding of ATP, induces an extensive conformational change that allows association of the binary complex with GroES. We modeled the epical domain movement from the GroEL monomer (modeled from chain A of PDB code 1SS8) to the GroEL-GroES-ADP7 monomer (modeled from chain A of PDB code 1SX4). Each symmetric complex was generated by applying 6 rotational transformations to the monomers to generate the 7-membered complex while imposing symmetry. The monomer contains 525 amino acids, and our model contains 13 rigid elements where most of the equatorial domain, whose structure does not change significantly, was modeled as one large segment and was considered fixed. The distance measure threshold for successful termination of the algorithm was a normalized distance of 0.18 from the goal conformation. The initial lRMSD between the C_α_ atoms of the two complexes is 12.21Å. Table 1 shows that our method significantly outperforms random walk both in runtime and average lRMSD. The average lRMSD between the resulting structures and the goal structure was 4.67Å compared to 6.11Å for MC. Many runs produced low lRMSD results in the order of magnitude of 3–4Å RMSD or less from the goal structure. The average run time was approximately 6.5 hours.

#### Analysis of the results

**Figure 3:**
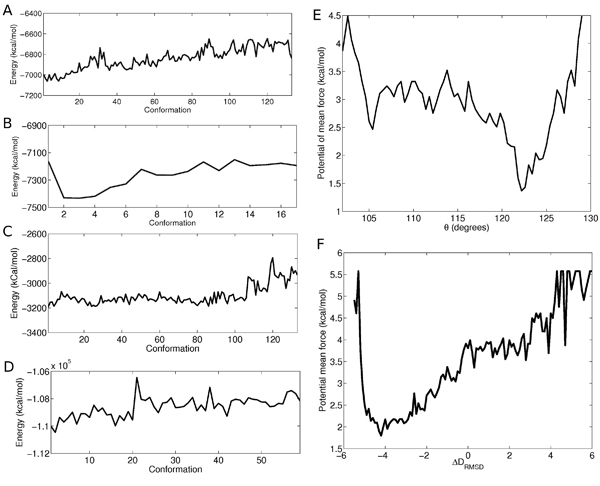
**Energetic profiles of the resulting pathways** Potential energy for ADK (A), RBP (B), GroEL (C) and CVN (D) along slightly minimized conformational pathways. Notice the different potential energy scale and different path lengths. Free energy along the Δ*D_rmsd_* reaction coordinate of the AdK pathway (E) and along the *θ* reaction coordinate for the RBP (F) pathway. See Results section for the definition of the reaction coordinates.

**Potential energy measurement** In order to provide initial validation for our results, we tested whether our algorithm produces biologically reasonable, low-energy pathways when using an all-atom force field. Such an analysis was done in an earlier work [28], where the authors used a similar method to show that their conformational search was reasonable. Side chain information was completed for the resulting pathways using the algorithm described in [42]. The resulting full-atomic structures were minimized for 1000 Steepest Descent steps using the AMBER energy minimization package [2] and subject to a harmonic restraining force of 10 kcal/mol/Å^2^. The minimization was done for a relatively small number of steps and was restrained in order to resolve initial clashes but not cause large conformational changes to the structures. The purpose of this test is not to provide a fully minimized pathway, but to show that the algorithm produces pathways with reasonable conformations whose clashes can be resolved within a small number of minimization steps. Figure 2 shows an example of a pathway for AdK, RBP, CVN, and GroEL and Figure 3(a)-(d) shows the potential energy plots of the corresponding pathways. In each case, the pathway chosen for figures 2 and 3(a)-(d) corresponds to the run with the lowest final lRMSD from the goal structure. For clarity, the conformations shown in the figures were sampled at approximately 1 distance measure unit from one another (see Distance Between Structures section for definition). As seen, even with a small number of energy minimization steps all the intermediate structures exhibit low potential energies, below —6000 kcal/mol for AdK, below —7000 kcal/mol for RBP, approximately —3000 kcal/mol for CVN and below —100,000 kcal/mol for GroEL, as measured by AMBER.

**Free energy profile for AdK** To provide further evidence that the produced paths are reasonable, we refer to a study [43] which provided an extensive analysis of the conformational pathway of AdK. The authors generated a conformational pathway using a Nudged Elastic Band (NEB) simulation [15]. Their large-scale analysis of the pathway included a free energy profile using umbrella sampling over a number of reaction coordinates. One of the reaction coordinates used for the free energy calculation was Δ*D_RMSD_* which is defined, given conformation *C*, as:

Δ*D_RMSD_*(*C*) = *RMSD* (*C*, *C_open_*) — *RMSD* (*C*, *C_closed_*)  (4)

We characterized the free energy profile along this reaction coordinate using our results. The data points were obtained by running the algorithm on AdK for 200 times. For each resulting pathway we recorded the Δ*D_RMSD_* value for the conformations along the pathway. To generate sets of uncorrelated conformations as required for free energy calculations, we sampled each pathway in spaces of 1 distance unit (see definition of the distance measure in the Methods section). Overall approximately 7500 conformations were included in the calculation. The free energy was calculated along the Δ*D_RMSD_* reaction coordinate using the Weighted Histogram Analysis Method (WHAM) [44]. It should be noted that the calculation was carried out under a number of assumptions: we used only backbone and C*_β_* and a relatively small number of samples. Therefore, our "pseudo free energy" results should be interpreted with caution. Also, our sampling method and potential of mean force calculation parameters differ significantly from the ones used in [43]. For these reasons, we can expect only qualitative similarity to the free energy profile obtained by that work and the absolute free energy values do not have the same meaning. The free energy profile shown in Figure 3(e) exhibits a qualitatively similar pattern to that shown in Figure 2(a) in [43] for the free conformational pathway: high free energy around a Δ*D_RMSD_* of 3 to 6 (closed conformation), and a low energy basin around the open conformation, at Δ*D_RMSD_* of –5 to –4. The spikes shown in the profile are the result of a relatively small number of samples and non-uniform sampling at some areas in the search space, whereas NEB provides an initially uniform interpolation. These results show that the sampling the algorithm provides along the conformational pathway is qualitatively similar to the one provided by NEB.

**Free energy profile for RBP** To provide further validation of our results we compare with another study which analyzed RBP [45]. The authors simulated the opening motion of the RBP protein and characterized the free energy profile using the reaction coordinate *θ*, which is the angle between the two domain, defined as the angle formed by the following three points: the center-of-mass (CM) of the N-terminal domain, the CM of the C-terminal domain and the CM of the hinge. The values of *θ* are 109 and 130 in the closed and open conformation, respectively. Our free energy calculations as a function of *θ* were conducted in a similar manner to the calculations described above for ADK. The result is shown in Figure 3(f) . Two minima are shown: one local minimum around 106 degrees and one global minimum at 123 degrees, very similar to the pattern shown in Figure 3(a) of [45]. It should be noted that we did not simulate the RBP mutant pathway discussed in [45], and therefore our plot ends at approximately 130 degrees.

In general, knowledge about intermediate states is needed in order to provide a case-specific validation, but this knowledge does not always exist. With the advances in structural detection and simulation methods, one can expect to have more information about intermediate states in the future. It should be noted that several intermediate structures already exist for AdK and a recent study makes use of those structures to validate their low energy profile calculations [46]. This is an important way to validate computational results and is the subject of present and future work. In cases where such information is not available, this algorithm can be viewed as an efficient initial filtering tool that reduces the tremendously high-dimensional space of possible conformations into a relatively small number of possible pathways. Refinement can then be made by other tools or indirect experimental knowledge to select biologically feasible pathways out of these possibilities. In the future we plan to apply clustering methods on the resulting pathways to extract more knowledge about feasible conformations and gain insight about the likelihood of each conformation along the resulting pathways.

**AdK intermediate result analysis** AdK has several known mutants and intermediate structures. In a recent study [46] the energy profile of AdK was produced using elastic network interpolation (ENI). The method was used to generate the conformational transition pathway between the open and closed form of AdK and compare the intermediates to known structural intermediates. Inspired by that study, we performed a similar test on our results. We focused on four known intermediates: chains A, B, and C of the hetero-trimer Adenylate Kinase from Aquifex Aeolicus (PDB accession code 2RH5), which are conformational change intermediates of the ligand free AdK [47] and 1E4Y, which is an AdK mutant having 99% sequence identity with 4AKE and 1AKE and is a closed form of AdK binding with AP5A. We selected our best 20 paths in terms of RMSD from the goal structure, all below 2.5Å, and recorded for each path the closest conformation to 1E4Y and to chains A, B and C of 2RH5. Our results are shown in Table 2. For each intermediate, the table shows the average RMSD from the closest conformation along the 20 paths and the conformation number (normalized to 1-100 to compare with the results in [46]). Our results are in good qualitative agreement with that work, which predicted 2RH5A-C and 1E4Y to be closest to the 88-100, 76-87, 68-78 and 1-12 percentiles, respectively (notice that in [46] the authors calculated the reverse path, from 4AKE to 1AKE).

**Table 2:** Comparison of our paths with intermediate structures of AdK

	**2RH5A**	**2RH5B**	**2RH5C**	**1E4Y**
	
lRMSD (Å)	2.55	2.47	2.96	2.82
Closest conformation (percent)	86.89	82.46	73.4	4.95

The closest pathway index (normalized to 1–100) and the lRMSD of the closest point to each PDB structures shown in Table 1 are listed. 2RH5 is from a hyperthermophilic E. coli and 1E4Y is an AdK mutant having 99% sequence identity with 1AKE and 4AKE. The measurements were taken over an average of 20 runs

**CVN path analysis** We compared 25 paths generated by our algorithm against a consensus path obtained by Raveh et al. [28] (B. Raveh, personal communication). We selected from our paths the ones yielding the closest RMSD to the goal structure, all below 2.5Å. We compared each one of our paths to the consensus path conformation-wise, recording the RMSD between each conformation along our path to its nearest neighbor along the consensus path. The paths tend to be similar towards the ends and deviate in the middle. The farthest point between our paths and the paths generated in [28] ranges between 5.9–10Å with an average of 8Å. The average distance between the endpoints of the paths is 3.15Å. The starting points are nearly identical between the two methods since both started from the same file. This is expected since the paths were obtained using different methods and different constraints. However, the fact that the differences between the paths were not very large in the edges of the paths and only deviated in the middle and even then not drastically on average, shows that the two methods are able to achieve similar results.

## Conclusions

We present a prototype for a novel method for exploring large scale conformational changes in proteins represented at the backbone level, requiring relatively little information. The search methodology is based on robot motion planning, and it strikes a balance between an efficient coverage of the conformational space and fast exploration towards the goal structure. A relatively simple potential function is used to guide the search. This representation and potential function make the computation tractable and especially useful in cases where side chain information is missing or if a detailed search is computationally infeasible. The goal of this paper is to provide an initial proof of concept for our method. Therefore, we tested our algorithm on the following four well studied proteins: Adenylate Kinase, Ribose binding protein, Cyanovirin N, and the GroEL complex. We show that our method performs significantly better than random walk by producing low energy pathways with resulting structures closer to the goal structure. We believe this is an important first step towards a larger scale modeling of more complex biological systems.

## Competing interests

The authors declare that they have no competing interests.

## Authors' contributions

All authors conceived the study. NH implemented the algorithm and performed the simulations. NH, MM, WC, and LEK drafted the manuscript. All authors read and approved the final manuscript.
